# Sleep-Disordered Breathing in Acute Stroke: A Single-Center, Prospective, Longitudinal Study

**DOI:** 10.3390/jcm12030986

**Published:** 2023-01-27

**Authors:** Panagiotis Plomaritis, Aikaterini Theodorou, Konstantinos Lourentzos, Maria-Ioanna Stefanou, Lina Palaiodimou, Georgia Papagiannopoulou, Vasiliki Kotsali-Peteinelli, Marianna Bregianni, Georgios P. Paraskevas, Georgios Tsivgoulis, Anastasios Bonakis

**Affiliations:** 1 Second Department of Neurology, “Attikon” University Hospital, School of Medicine, National and Kapodistrian University of Athens, 12462 Athens, Greece; 2 Department of Neurology, University of Tennessee Health Science Center, Memphis, TN 38163, USA

**Keywords:** acute stroke, sleep-disordered breathing, polysomnography, predictors, functional outcome, mortality, stroke severity, stroke subtype

## Abstract

Background: Sleep-disordered breathing (SDB) is common among acute stroke patients. We sought to investigate the prevalence, severity and type of SDB in consecutive acute stroke patients. Moreover, we aimed to identify independent predictors of SDB in the acute stroke setting and investigate potential associations between SDB and functional outcomes at three months. Methods: We prospectively studied consecutive acute stroke patients, who underwent overnight polysomnography within 72 h from symptom onset. Demographics, clinical and imaging characteristics were documented. Daytime sleepiness preceding the stroke, stroke severity on admission and functional outcome at three months were evaluated using the Epworth-Sleepiness Scale (ESS), National Institute of Health Stroke Scale (NIHSS) and modified Rankin Scale (mRS), respectively. SDB was documented using standard polysomnography criteria. Results: A total of 130 consecutive acute stroke patients were prospectively evaluated [110 with ischemic stroke and 20 with intracerebral hemorrhage, mean age 60.5 ± 10.9 years, 77% men, median NIHSS score on admission: 3 (IQR: 2–17)]. The rate of SDB detection on polysomnography recordings was 79% (95% CI: 71–86). Three variables were independently associated with the likelihood of SDB detection in multivariable analyses adjusting for potential confounders: age (OR per 10-year-increase: 2.318, 95% CI: 1.327–4.391, *p* = 0.005), male sex (OR: 7.901, 95% CI: 2.349–30.855, *p* = 0.001) and abnormal ESS-score (OR: 6.064, 95% CI: 1.560–32.283, *p* = 0.017). Among patients with SDB, congestive heart failure was independently associated with the likelihood of central apnea detection (OR: 18.295, 95% CI: 4.464–19.105, *p* < 0.001). Among all patients, increasing NIHSS score on admission (OR: 0.817, 95% CI: 0.737-0.891, *p* < 0.001) and Apnea–Hypopnea Index (OR: 0.979, 95% CI: 0.962–0.996, *p* = 0.020) emerged as independent predictors of excellent functional outcome at 3 months (mRS-scores 0–1). Conclusion: The high prevalence and severity of SDB in acute stroke patients and its negative impact on functional outcome indicate the importance of polysomnography implementation in everyday clinical practice of acute stroke work-up and management.

## 1. Introduction

Stroke is the leading cause of disability and the second leading cause of death worldwide [1]. Globally, there are over 101 million people who have survived stroke [2]. The presence of modifiable risk factors allows both primary and secondary prevention of stroke. The traditional stroke risk factors are hypertension, diabetes mellitus, dyslipidemia, smoking, atrial fibrillation, high body mass index (BMI), physical inactivity and carotid stenosis [3]. 

Sleep-disordered breathing (SDB) is a common chronic medical condition affecting 936 million people globally, almost half of which have moderate to severe sleep apnea [4]. The prevalence of SDB in the general population is higher in men, in older ages and in those with higher BMI [5]. Sleep apnea is characterized by repeated episodes of partial (hypopnea) or complete (apnea) cessation of airflow occurring during sleep [6,7]. These episodes can cause intermittent hypoxia and arousals resulting in sleep fragmentation and symptoms such as daytime sleepiness, fatigue, irritability and impaired concentration, which constitute sleep apnea syndrome (SAS). SDB includes three different types, obstructive sleep apnea (OSA), central sleep apnea (CSA) and mixed sleep apnea. The gold standard diagnostic method for identifying SDB is polysomnography (PSG). SDB is defined by an apnea–hypopnea index (AHI) greater than five events per hour of sleep. According to AHI, SDB is classified as mild (5 ≤ AHΙ < 15), moderate (15 ≤ AHI < 30) or severe (AHI ≥ 30) [6,7].

It is well established that SDB increases the risk of stroke both directly as an independent factor and indirectly by affecting other vascular risk factors such as hypertension and arrythmias [8,9,10,11]. On the other hand, acute stroke is associated with certain complications, such as disturbed coordination of upper airway, prolonged supine position, sleep fragmentation and brainstem respiratory center damage, which can generate de novo sleep apnea [6,12]. Multiple studies have shown a high prevalence of SDB among acute stroke patients which appears to be approximately 70% [13]. OSA is the predominant type of SDB after stroke, while the prevalence of CSA is only 12% [10,13]. Current evidence shows that the presence of SDB is associated with poor outcome and reduced likelihood of functional independence at three months after stroke [14,15]. Moreover, OSA is a risk factor for stroke recurrence and may be associated with an increase in all-cause mortality after stroke [10,16,17]. Treatment of OSA patients with continuous positive airway pressure (CPAP) seems to reduce the risk of stroke and improve neurological recovery after stroke [18,19,20,21,22,23]. 

The aim of this prospective observational study was to provide an estimate of the prevalence and severity of SDB in acute stroke patients in Greece and to evaluate its potential associations with severity, subtype and etiology of stroke, demographic characteristics, vascular risk factors and functional outcomes at 3 months. 

## 2. Materials and Methods

The data that support the findings of the present study are available from the corresponding author on reasonable request. This study was performed in accordance with the STROBE guidelines for reporting observational research [24].

### 2.1. Study Design and Regulations

We performed a single-center, prospective, observational study. Institutional review board approval was obtained prior to all study-related activity from the ethics committee of “Attikon” University Hospital (Decision Number: ΕΒD113/27-02-2018). Written informed consent was obtained from all patients or their legal representatives before enrollment.

### 2.2. Setting and Eligibility Criteria

The study was conducted at the Second Department of Neurology of the National and Kapodistrian University of Athens located at “Attikon” University Hospital, Athens, Greece. This is a tertiary care stroke center covering the western part of the Attica region [25,26,27]. We prospectively enrolled consecutive adult (≥18 years) patients with acute ischemic stroke (AIS) or intracerebral hemorrhage (ICH) during a four-year period (May 2018–September 2022), who underwent overnight PSG within 72 h from stroke onset.

Patients were excluded if they were diagnosed with transient ischemic attack or if they presented with severe stroke symptoms (stupor/coma, or NIHSS score >25 points), aphasia with poor comprehension or acute confusional state with or without agitation that would render cooperation with PSG unfeasible. Patients with acute respiratory infection, baseline oxygen saturation <95%, stroke mimics including seizures and postictal paralysis, toxic–metabolic disturbances and brain tumors were also excluded to rule out confounders that could interfere with measurements [28]. Patients who did not provide consent to undergo PSG or participate in the current study were also excluded. Data from PSG were judged to be non-interpretable, resulting in further exclusion of patients from the current study, if at least one of the following criteria was fulfilled: (1) >70% of the data were lost in the recording, (2) >80% of total recording time had a poor airflow signal, (3) impossible evaluation of sleep stage, and (4) insufficient total sleep time (≤3 h) [29].

### 2.3. Data Collection

Data on demographics and clinical characteristics, including cardiovascular risk factors were collected as previously described [25,26,27]: (1) demographic characteristics: age, sex; (2) vascular risk factors: BMI/obesity, hyperlipidemia, arterial hypertension, diabetes mellitus, atrial fibrillation, congestive heart failure, presence of intracardiac thrombus or mechanical valve, history of stroke or TIA, history of myocardial infarction, current smoking, history of excessive alcohol intake; (3) acute reperfusion therapies in AIS; (4) stroke severity assessed by NIHSS score (National Institute of Health Stroke Scale) on admission (NIHSS_adm_) and at hospital discharge (NIHSS_dis_) [30]; (5) and functional clinical outcome assessed by mRS (modified Rankin Scale) score at 3 months post index event [31].

All patients underwent comprehensive diagnostic work-up during hospitalization in accordance with the American Heart Association (AHA) recommendations [32], including (1) brain computed tomography (CT) or magnetic resonance imaging (MRI) scan with CT- or MR-angiography of cervical and cerebral vessels; (2) ultrasonography of the cervical and cerebral arteries performed by a certified neurosomnologist; (3) transthoracic or transoesophageal echocardiogram, a 12-lead electrocardiogram (ECG), and/or an ECG Holter monitoring (>24 h) performed by certified cardiologists; (4) routine blood tests/laboratory investigations, as standard of care. Details about the diagnostic work-up in our tertiary stroke center have been previously published [25,26,27].

All patients were classified according to discharge diagnosis as AIS or ICH, and classification of ischemic stroke etiology was made according to the TOAST (Trial of ORG10172 in Acute Stroke Treatment) criteria [33]. We also used recent diagnostic criteria to identify patients with embolic stroke of undetermined source (ESUS) as previously described [34]. All patients were prospectively followed-up, and the clinical outcome 90 days after the index event was captured as follows: excellent functional outcome (mRS scores of 0–1) versus poor functional outcome (mRS scores of 2–6) within 90 days after index event. All outcome events were assessed by attending-level stroke neurologists who were blinded to the PSG findings.

### 2.4. Sleep Evaluation

Daytime sleepiness preceding the stroke was evaluated in every patient by means of a questionnaire using the Epworth Sleepiness Scale (ESS) and was considered abnormal when the score was >9 [35,36]. All patients underwent a type 2 (unattended) overnight sleep study at the hospital wards from 10 pm to 8 am. For this purpose, a Nox-A1 PSG ambulatory monitoring system (Nox Medical, Inc., Reykjavik, Iceland) was used, which consists of six electroencephalogram (EEG) channels (F3-M2, F4-M1, C3-M2, C4-M1, O1-M2 and O2-M1), 2 electrooculogram (EOG) channels and 3 submental electromyogram (EMG) channels for sleep stage evaluation, 2 anterior tibialis EMG channels for limb movement scoring, an electrocardiogram, a nasal airflow/pressure sensor, respiratory inductive plethysmography, body position sensor and wireless pulse oximetry.

According to the available literature, apnea was defined as a 90% or greater decrease in airflow compared with preceding signals for a minimum of 10 s [37]. An obstructive apnea was associated with the evidence of continued respiratory effort throughout the event and a central apnea was associated with the absence of respiratory effort throughout the event. Mixed apneas were associated with an absence of respiratory effort during the initial part of the event, followed by the appearance of respiratory effort during the latter part of the event [37,38].

Hypopnea was defined as at least a 30% decrease in airflow compared with the pre-event baseline, lasting at least 10 s and associated with either a 3% oxygen desaturation from baseline or an electroencephalography (EEG) arousal [37]. Apnea–hypopnea index (AHI) was defined as the combined number of apneas and hypopneas that occurred per hour of sleep and SDB was diagnosed based on AHI ≥5. According to AHI, sleep apnea was classified as mild when 5 ≤ AHI < 15, moderate when 15 ≤ AHI < 30 and severe when AHI ≥ 30 [37]. SDB was considered as OSA or CSA if >50% of the respiratory events were of obstructive or of central origin, respectively.

Sleep stages and apnea/hypopnea events were scored manually by an experienced neurosomnologist (AB) according to the American Academy of Sleep Medicine scoring criteria (AASM, scoring manual version 2.6, 2020) [7]. 

### 2.5. Statistical Analysis

Continuous parametric data were presented using their mean values together with their corresponding standard deviations (SDs), whereas median values with their corresponding interquartile ranges (IQR) were used for the presentation of nonparametric data. Categorical variables are presented as percentages with their corresponding 95% confidence intervals (95% CI). Statistical comparisons between two groups were performed using a chi-square test, or in case of small, expected frequencies, Fisher’s exact test. Continuous variables were compared by the use of the unpaired *t*-test or Mann–Whitney U test, as indicated. 

Univariable and multivariable binary logistic regression models were used to evaluate associations between baseline characteristics with the likelihood of detecting SDB among patients with acute ischemic or hemorrhagic stroke, to evaluate associations between baseline characteristics with the likelihood of detecting central versus obstructive sleep apnea among patients with SDB, and to evaluate associations between baseline characteristics with the likelihood of excellent functional clinical outcome at 3 months (defined as mRS-scores of 0–1) among all patients and among SDB patients, before and after adjusting for potential confounders. A cutoff of *p* < 0.1 was used to select variables for inclusion in multivariable analyses that were conducted using a backward stepwise selection procedure. To confirm the robustness of the multivariable models, we repeated all multivariable analyses using a forward selection procedure. Associations are presented as odds ratios (OR) with corresponding 95% confidence intervals (CI). Statistical significance was achieved if the *p* value was ≤0.05 in multivariable logistic regression analyses. The Stata/SE Statistical Software Release 13 for Mac and the R—software version 3.5.0 were used for statistical analyses [39].

## 3. Results

During the four-year study period, we included a total of 130 consecutively admitted patients within 72 h from symptom onset [mean age 60.5 ± 10.9 years, 77% men, median NIHSS score on admission:3 (IQR: 2–17)] with AIS (*n* = 110, 85%) or ICH (*n* = 20, 15%), who fulfilled our inclusion criteria. Baseline characteristics of the study population, including admission NIHSS scores, cardiovascular risk factors, underlying etiologies of the qualifying event in accordance with the TOAST classification, results of the diagnostic work-up during hospitalization and acute reperfusion therapies, are summarized in Table 1. In the acute phase, the overall rate of sleep apnea was 79% (95% CI: 71–86), the rate of sleep apnea among patients with AIS was 82%, and among patients with ICH it was 65%. The majority of patients with SDB were men (76%). The rates of mild, moderate and severe SDB were 16%, 24% and 60%, respectively, and the rates of obstructive and central apnea were 81% and 19%, respectively. The mean AHI was 33.5 ± 24.8.

The univariable and multivariable associations of baseline characteristics and the likelihood of sleep apnea detection are presented in Table 2. The following variables were associated with sleep apnea detection on initial univariable analyses using a *p*-value of <0.1 as threshold for inclusion in multivariable models: increasing age, male sex, obesity (BMI > 30 kg/m^2^), ICH subtype, diabetes mellitus, arterial hypertension, hyperlipidemia and excessive daytime sleepiness, which was assessed as a score >9 using the Epworth Sleepiness Scale. The following three variables were independently (*p* < 0.05) associated with the likelihood of sleep apnea detection in the acute stroke phase in multivariable logistic regression analyses conducted by backward selection procedure: age (OR per 10-year increase: 2.318, 95% CI: 1.327–4.391, *p*-value: 0.005), male sex (OR: 7.901, 95% CI: 2.349–30.855, *p*-value: 0.001) and abnormal ESS score (OR: 6.064, 95% CI: 1.560–32.283, *p*-value: 0.017). We repeated the multivariable analyses using the forward selection procedure and obtained identical results. 

With respect to clinical outcome, all patients were followed for 3 months and independent predictors of excellent functional outcome were increasing NIHSS (OR per 1 point increase: 0.817, 95% CI: 0.737–0.891, *p* < 0.001) and increasing Apnea–Hypopnea Index (OR per 1 point increase: 0.979, 95% CI: 0.962–0.996, *p* = 0.020) (Table 3).

The univariable and multivariable associations of baseline characteristics and the likelihood of central sleep apnea detection among patients with SDB are presented in Table 4. The following variables were associated with central sleep apnea detection on initial univariable analyses using a *p*-value of <0.1 as threshold for inclusion in multivariable models: increasing BMI, increasing NIHSS score, congestive heart failure, history of myocardial infarction, excessive alcohol intake and ESUS (vs. non-ESUS) stroke subtype. Only congestive heart failure (OR: 18.295, 95% CI: 4.464–19.105, *p* < 0.001) was independently associated with the likelihood of central sleep apnea detection in the acute stroke stage in multivariable logistic regression analyses conducted by backward selection procedure (Table 4). We repeated additionally the multivariable analyses using the forward selection procedure and obtained identical results.

## 4. Discussion

Our study shows a high prevalence of SDB in the acute stroke setting with 79% and 60% of consecutive acute stroke patients having an AHI ≥ 5/h (corresponding to SDB) and AHI ≥ 30/h (corresponding to severe SDB). Increasing age, male sex and abnormal ESS score were independently associated with higher odds of SDB. OSA was the predominant type of sleep apnea in acute stroke stage (81%). Moreover, we found that congestive heart failure was strongly associated with CSA detection after stroke. Finally, our study results suggest that both increasing NIHSS score on admission and increasing AHI estimated at the first 72 h after stroke onset are significant predictors of poor functional outcome at three months.

The 79% prevalence of SDB in our study is higher than the one (67–75%) estimated in stroke patients in other prospective studies and the most recent meta-analysis [13,40,41,42,43]. We believe that this difference can be attributed to the following facts. First, our study focused exclusively on the hyperacute phase of stroke (within 72 h from stroke onset). Previous studies and a recent meta-analysis showed that the prevalence of moderate and severe SDB seems to decrease from acute to chronic phases of stroke [14,40,43,44]. Moreover, the use of full PSG with EEG in our study allowed the identification of arousals, which in combination with an AHI cutoff of 5/h for SDB diagnosis and the use of 3% threshold for the definition of hypopnea, increased the sensitivity of SDB detection. 

The predominance of male sex and OSA type in stroke patients with SDB in our study is in line with the findings of previous reports [13,45,46]. Furthermore, the rate of CSA (19.4%) is within the range of percentages (6–23%) reported in the most recent meta-analysis [13]. As formerly reported in the literature, no significant difference regarding the prevalence of SDB between ischemic and hemorrhagic stroke was documented in our Greek cohort [45], indicating that stroke pathophysiology has no effect on risk of SDB.

Independent predictors of SDB in stroke patients have not been previously investigated in detail [10]. Some studies performed in the past showed increasing age, BMI, male sex, diabetes mellitus, smoking and increasing NIHSS as risk factors for SDB after stroke [14,40,47,48]. However, these reports failed to adjust for important confounders and did not take into account the underlying ischemic stroke etiology. Moreover, previous studies did not show any significant association between excessive daytime sleepiness (assessed by ESS) and sleep apnea in stroke patients [40,49]. Nevertheless, our study demonstrated that apart from increasing age (per 10-year increase) and male sex, abnormal ESS score can also predict the presence of SDB in patients with acute stroke. This result appears conflicting with the findings of previous reports but differences in study populations, confounders included in the multivariable analyses, time frame of PSG in the acute stroke stage and ethnic differences may account for these discrepant results. 

Although obesity (defined as ΒΜΙ >30) is a well-known risk factor for OSA in the general population, our analysis did not manage to demonstrate obesity as a significant predictor of SDB among acute stroke patients. A possible reason for that could be the small size of our sample and the respectively small number of obese patients included. However, in our study there was a marginally significant association between increasing BMI and the likelihood of detecting OSA among stroke patients with SDB (Table 4).

There is evidence supporting the association of SDB with either macroangiopathy (large artery atherosclerosis) or cardioembolism as cause of ischemic stroke [40,50,51]. In the present study, no relationship between SDB and stroke etiology was detected. This finding is in line with the results of the BASIC sleep apnea study [52], but requires additional confirmation in a larger multi-center setting. Additionally, we confirm that stroke severity and brainstem stroke localization do not predict the presence of SDB [40,53,54]. With regard to SDB type, our study showed that congestive heart failure is strongly associated with the detection of CSA in the setting of stroke which lies in contrast to the results of previous studies in stroke patients [14,55]. This finding can be explained if we take into account the fact that congestive heart failure is strongly related to the Cheyne–Stokes type of CSA [56]. 

Our analysis for excellent functional outcome at 3 months in patients with acute stroke demonstrated that both increasing stroke severity and SDB severity (assessed by NIHSS and AHI respectively) are independent predictors of poor functional outcome. Our results are supported by three previous prospective observational studies which also used mRS for stroke outcome assessment [14,15,57]. 

There are certain limitations that should be taken into account when interpreting our study results. First, our sample size was small and our study population had certain characteristics that may have introduced selection bias. In particular, the NIHSS score on admission was rather low compared to the one of unselected AIS patients admitted in our tertiary stroke center during the study period (median NIHSS score 3 versus 7, respectively). Moreover, there was a high prevalence of male patients and a lower rate of ICH subtype. In addition, patients with high stroke severity, aphasic comprehensive disorders, confusion or agitation were excluded. Therefore, our results may not be representative of all stroke patients. Finally, our data acquisition did not entail recording of history of habitual snoring or neck circumference that may have influenced the reported association between SDB severity and poor functional outcome at three months. However, we used a full PSG study to detect SDB in 130 patients during the hyperacute stroke phase and the recordings were afterwards evaluated by experienced sleep investigators. This represents an important strength of the current study in view of the following considerations: (a) the relative clinical instability and poor cooperation of patients during the first 72 h from stroke onset, (b) the difficulty of placing a full PSG setting on patients in hospital wards and (c) the possibility of misinterpreting the PSG results by single automatic scoring of sleep stages and respiratory events. 

In conclusion, SDB prevalence in Greek patients with acute stroke is very high with the majority of them having severe sleep apnea and OSA type. Predictors of SDB presence in the acute stroke phase are increasing age, male sex and excessive daytime sleepiness. Congestive heart failure is strongly associated with the detection of CSA after stroke. Finally, increasing stroke severity and SDB severity are independent predictors of poor functional outcome at three months after stroke. The high prevalence and severity of SDB in acute stroke patients and its negative impact on functional outcome indicate the importance of polysomnography implementation in everyday clinical practice of acute stroke work-up and management. 

## Figures and Tables

**Table 1:** Baseline characteristics of the study population (*n* = 130).

Variable	Overall
Age (years), mean (SD *)	60.5 (10.9)
Sex—Male, *n* (%)	88 (77.0%)
BMI, mean (SD *)	29.3 (5.7)
Obese, *n* (%)	48 (36.9%)
Intracerebral Hemorrhage, *n* (%)	20 (15.4%)
Ischemic Stroke, *n* (%)	110 (84.6%)
Acute reperfusion therapies, *n* (%)	16 (14.5%)
**Neurologic Deficit**	
NIHSS_adm_– Score, median (IQR **)	3 (2–7)
NIHSS_dis_– Score, median (IQR **)	2 (0–3)
**mRS at Discharge**	
excellent—poor	77 (59.2%)–53 (40.8%)
**mRS at 3 months**	
excellent—poor	89 (68.4%)–41 (31.5%)
**Comorbidities**	
Hyperlipidemia, *n* (%)	49 (37.7%)
Arterial Hypertension, *n* (%)	81 (62.3%)
Diabetes Mellitus, *n* (%)	32 (24.6%)
Congestive Heart Failure (CHF), *n* (%)	12 (9.2%)
History of Stroke/Transient Ischemic Attack, *n* (%)	22 (16.9%)
History of Myocardial Infarction, *n* (%)	25 (19.2%)
PFO, *n* (%)	7 (5.4%)
Atrial Fibrillation, *n* (%)	20 (15.4%)
Reduced Ejection Fraction +/− Dilated Left Atrium, *n* (%)	3 (2.3%)
Intracardiac Thrombus, *n* (%)	1 (<1%)
Mechanical Valve, *n* (%)	1 (<1%)
Current Smoking, *n* (%)	67 (58.4%)
Excessive Alcohol Intake, *n* (%)	14 (10.8%)
**TOAST—Classification**	
Non Cryptogenic, *n* (%)	79 (60.8%)
Cryptogenic ESUS, *n* (%)	33 (25.4%)
Cryptogenic non-ESUS, *n* (%)	18 (13.8%)
**Sleep-disordered breathing**	
**Epworth—Sleepiness Scale score**	
0–9 points, *n* (%)	58 (59.2%)
≥10 points, *n* (%)	40 (40.8%)
**Sleep-disordered breathing—SDB, *n* (%)**	103 (79.2%; 95% CI: 71.2—85.8)
Sex—Male, *n* (%)	78 (75.7%)
Ischemic Stroke, *n* (%)	90 (81.8%)
Acute reperfusion therapies, *n* (%)	14 (87.5%)
Apnea—Hypopnea Index (AHI), mean (SD *)	33.5 (24.8)
**Classification of SDB**	
Mild, *n* (%)	16 (15.5%)
Moderate, *n* (%)	25 (24.3%)
Severe, *n* (%)	62 (60.2%)
**Type of SDB**	
Obstructive, *n* (%)	83 (80.6%)
Central, *n* (%)	20 (19.4%)

* SD: Standard Deviation, ** IQR: Interquartile Range.

**Table 2:** Univariable and multivariable logistic regression analyses depicting the associations of baseline characteristics with the likelihood of detecting sleep-disordered breathing (SDB).

Variable	Univariable Logistic Regression Analysis		Multivariable Logistic Regression Analysis	
	Odds Ratio (95% CI)	*p*-Value *	Odds Ratio (95% CI)	*p*-Value *
Age	1.621 (1.082–2.493)	0.022	2.318 (1.327–4.391)	0.005
(per 10-year increase)				
Male (Sex)	5.304 (2.191–13.485)	<0.001	7.901 (2.349–30.855)	0.001
Obese (ΒΜΙ > 30 Kg/m^2^)	4.288 (1.517–15.411)	0.012	3.450 (0.743–20.787)	0.136
ICH vs. AIS	0.413 (0.148–1.217)	0.095	0.229 (0.036–1.420)	0.109
NIHSS—Score	1.059 (0.973–1.179)	0.232		
(per 1-point increase)				
Hyperlipidemia	2.508 (0.982–7.306)	0.068	2.033 (0.370–14.410)	0.434
Arterial Hypertension	2.537 (1.075–6.116)	0.035	2.341 (0.686–8.332)	0.175
Diabetes Mellitus	3.135 (0.996–13.898)	0.079	0.306 (0.039–2.265)	0.240
Congestive Heart Failure	3.109 (0.564–58.127)	0.288		
History of Stroke/TIA	0.487 (0.179–1.415)	0.167		
History of Myocardial Infarction	2.173 (0.677–9.729)	0.238		
PFO	0.323 (0.067–1.729)	0.156		
Atrial Fibrillation	1.581 (0.480–7.171)	0.492		
Brainstem Stroke Location	1.854 (0.569–8.359)	0.352		
Current Smoking	1.088 (0.541–2.242)	0.815		
Excessive Alcohol Intake	3.756 (0.697–69.802)	0.212		
ESUS	1.726 (0.912–3.675)	0.119		
Epworth-Sleepiness-Scale	3.732 (1.246–13.886)	0.028	6.064 (1.560–32.283)	0.017
Score > 9				

* cutoff of *p* < 0.1 was used for selection of candidate variables for inclusion in multivariable logistic regression models.

**Table 3:** Univariable and multivariable logistic regression analyses depicting the associations of baseline characteristics with the likelihood of excellent functional outcome (mRS-score 0–1) at 3 months among all patients.

Variable	Univariable Logistic Regression Analysis		Multivariable Logistic Regression Analysis	
	Odds Ratio (95% CI)	*p*-Value *	Odds Ratio (95% CI)	*p*-Value *
Age	0.865 (0.608–1.218)	0.408		
(per 10-year increase)				
Male (Sex)	0.814 (0.356–1.792)	0.615		
Obese (ΒΜΙ > 30 Kg/m2)	0.755 (0.354–1.625)	0.467		
ICH vs. AIS	1.459 (0.519–4.769)	0.496		
NIHSS	0.820 (0.743–0.891)	<0.001	0.817 (0.737–0.891)	<0.001
(per 1-point increase)				
Hyperlipidemia	1.072 (0.501–2.338)	0.859		
Arterial Hypertension	0.582 (0.256–1.265)	0.181		
Diabetes Mellitus	1.018 (0.438–2.483)	0.968		
Congestive Heart Failure	0.914 (0.269–3.598)	0.888		
History of Stroke/TIA	2.345 (0.805–8.565)	0.148		
History of Myocardial Infarction	0.413 (0.168–1.018)	0.053		
PFO	0.596 (0.126–3.148)	0.512		
Atrial Fibrillation	1.089 (0.400–3.291)	0.872		
Reduced EF +/− Dilated LA	0.919 (0.086–20.125)	0.946		
Brainstem—Stroke	0.496 (0.194–1.287)	0.143		
Current Smoking	1.195 (0.647–2–246)	0.573		
Excessive Alcohol Intake	0.810 (0.260–2.793)	0.722		
ESUS	1.652 (0.961–3.022)	0.083	1.698 (0.927–3.304)	0.099
Sleep-disordered breathing (SDB)	0.423 (0.133–1.134)	0.109		
Central (vs. Obstructive) SDB	0.764 (0.282–2.149)	0.598		
Apnea–Hypopnea Index	0.982 (0.967–0.997)	0.022	0.979 (0.962–0.996)	0.020
Epworth-Sleepiness-Scale	0.839 (0.335–2.134)	0.708		
Score > 9				

* cutoff of *p* < 0.1 was used for selection of candidate variables for inclusion in multivariable logistic regression models.

**Table 4:** Univariable and multivariable logistic regression analyses depicting the associations of baseline characteristics with the likelihood of detection of central apnea vs. obstructive apnea among patients with sleep-disordered breathing (SDB).

Variable	Univariable Logistic Regression Analysis		Multivariable Logistic Regression Analysis	
	Odds Ratio (95% CI)	*p*-Value *	Odds Ratio (95% CI)	*p*-Value *
Age	0.897 (0.535–1.485)	0.672		
(per 10-year increase)				
Male (Sex)	2.044 (0.611–9.352)	0.289		
BMI	0.899 (0.793–1.000)	0.071	0.889 (0.768–1.005)	0.084
(per 1-point increase)				
ICH vs. AIS	0.311 (0.017–1.737)	0.277		
NIHSS	1.072 (0.986–1.163)	0.095	1.049 (0.939–1.173)	0.396
(per 1-point increase)				
Hyperlipidemia	0.703 (0.243–1.901)	0.497		
Arterial Hypertension	0.895 (0.327–2.618)	0.833		
Diabetes Mellitus	1.118 (0.359–3.161)	0.838		
Congestive Heart Failure	17.778 (4.486–90.359)	<0.001	18.295 (4.464–9.105)	<0.001
History of Stroke/TIA	0.598 (0.089–2.432)	0.523		
History of Myocardial Infarction	5.917 (2.041–17.646)	<0.001	2.995 (0.605–13.525)	0.157
Atrial Fibrillation	1.972 (0.561–6.232)	0.26		
Brainstem—Stroke	1.595 (0.459–4.924)	0.432		
Current Smoking	1.358 (0.601–3.076)	0.457		
Excessive Alcohol Intake	3.125 (0.847–10.766)	0.073	3.402 (0.710–15.661)	0.114
ESUS	0.461 (0.179–0.983)	0.068	0.609 (0.214–1.465)	0.301
Epworth-Sleepiness-Scale	0.665 (0.184–2.214)	0.513		
Score > 9				

* cutoff of *p* < 0.1 was used for selection of candidate variables for inclusion in multivariable logistic regression models.

## Data Availability

The data that support the findings of this study are available from the corresponding author (G.T.) upon reasonable request.
